# Approaching full cost recovery: the IAM NOOR eye care programme

**Published:** 2013

**Authors:** Melissa Diaz, Hans Limburg

**Affiliations:** International Public Health Consultant: MUNDIAZ Health Support, Amsterdam, The Netherlands. **md@mundiaz.com**; Consultant: Public Eye Health and Heath Information Services, Grootebroek, The Netherlands. **hlimburg@quicknet.nl**

**Figure:**
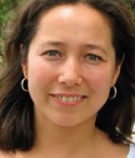
Melissa Diaz

**Figure:**
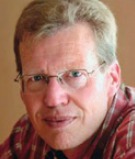
Hans Limburg

Eye care programmes can become more sustainable and less dependent on external funding by charging user fees and thereby generating revenue to cover operating costs. Ideally, a substantial surplus will be generated to cover the costs of free surgery for the poor and fund capital expenses and institutional growth.

Afghanistan is a country recovering from decades of war and instability. Even without war, Afghanistan's geography, climate and infrastructure constitute major challenges to providing adequate care to rural communities. Health indicators are amongst the worst in the world and blinding eye diseases are a significant public health problem.

The International Assistance Mission (IAM) is an international non-governmental organisation that has been implementing the IAM NOOR Eye Care Program in Afghanistan since 1966. IAM NOOR believes that high quality eye care services cannot be provided free of charge. The organisation also believes that services should be accessible for all persons in need, and it therefore provides a fee waiver for people who are unable to pay for treatment or surgery.

This article discusses an investigation conducted from 2011–2012 into the differences and similarities between two groups of hospitals in Afghanistan.

1. Four hospitals that are run by IAM NOOR.
2. Two government-run eye hospitals which have a contract with IAM NOOR that allows them to charge user fees. (Under the constitution, other governmental hospitals are not allowed to charge user fees.)

## Level of cost recovery

- The IAM NOOR-run hospital in Mazar-i-Sharif had nearly achieved full cost recovery (98%).
- The other three IAM NOOR hospitals still have substantial dependence on donor funding and have cost recovery of between 47% and 62%.
- Both government-run hospitals have nearly reached cost recovery of 88% and 89% respectively.

**Figure:**
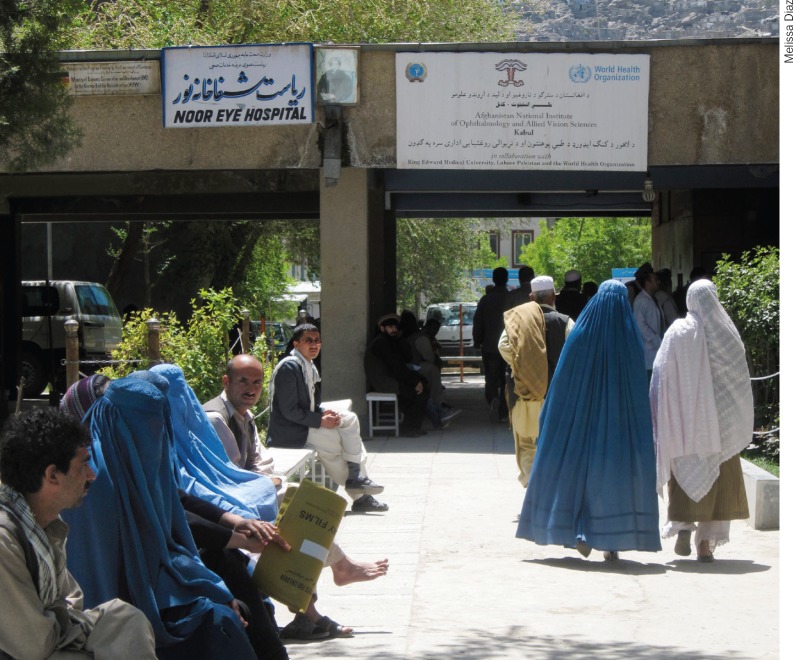
IAM NOOR Eye Hospital

## Reasons for level of cost recovery

In the IAM NOOR-run eye hospital in Mazar-i-Sharif, the high level of cost recovery was mainly due to the high patient numbers (which increased income) and good efficiency.

Lower levels of cost recovery in the other three IAM NOOR hospitals were partly due to the fact that some of these are only recently operational and patient numbers are low. Cost recovery is expected therefore to increase once patient numbers go up and the hospitals are able to recruit more paramedical eye care workers (which will improve efficiency). The aim is to reach a ratio of one eye surgeon to five paramedical staff members. Patient numbers could be increased by operating on cataracts at an earlier stage, e.g. in eyes with a visual acuity (VA) better than the current threshold of 6/60. Reducing all unnecessary routine laboratory screening will reduce the costs of the service, thereby attracting more patients.

In the two government-run hospitals, the high levels of cost recovery were due to having lower expenses. These hospitals had relatively low efficiency compared to the IAM NOOR-run hospital in Mazar-i-Sharif; however, they provided substantially lower salaries and implemented other cost-cutting measures, including lower maintenance expenses, which increased their cost recovery.

## Conclusion

Over the past decades, IAM NOOR has successfully provided eye care in Afghanistan, both through setting up and running eye care facilities and by supporting government eye hospitals through a partnership agreement. All facilities charge user fees to cover costs, and some clinics have nearly reached full cost recovery. Full cost recovery, however, does not necessarily imply that the facility is running at maximum capacity, nor that it is delivering high quality care to all patients in need of eye care. Nevertheless, cost recovery is essential to make eye care programmes sustainable in the long run.

### Author's note

*IAM NOOR notes that it is encouraged by the results in cost recovery, established in such a challenging context. It will use the outcome of this evaluation to further improve sustainability of its eye care services in the coming years*.

*The authors express their gratitude to both IAM NOOR and Light for the World Netherlands for the opportunity to perform this evaluation and for providing supportive services. For a copy of the full report, please email Klaas Aikes:* **k.aikes@lightfortheworld.nl**

